# Phase 1 study of DS‐1205c combined with gefitinib for EGFR mutation‐positive non‐small cell lung cancer

**DOI:** 10.1002/cam4.5508

**Published:** 2023-01-09

**Authors:** Koichi Goto, Yoshimasa Shiraishi, Haruyasu Murakami, Hidehito Horinouchi, Ryo Toyozawa, Masayuki Takeda, Makiko Uno, Nigel Crawford, Joseph McGill, Takeshi Jimbo, Masato Ishigami, Gensuke Takayama, Shintaro Nakayama, Shoichi Ohwada, Makoto Nishio

**Affiliations:** ^1^ National Cancer Center Hospital East Kashiwa Japan; ^2^ Kyushu University Hospital Fukuoka Japan; ^3^ Shizuoka Cancer Center Shizuoka Japan; ^4^ National Cancer Center Hospital Tokyo Japan; ^5^ National Hospital Organization Kyushu Cancer Center Fukuoka Japan; ^6^ Kindai University Hospital Osaka Japan; ^7^ Daiichi Sankyo Co. Ltd. Tokyo Japan; ^8^ Daiichi Sankyo Inc. Basking Ridge New Jersey USA; ^9^ Department of Thoracic Medical Oncology The Cancer Institute Hospital of Japanese Foundation for Cancer Research Tokyo Japan

**Keywords:** AXL inhibitor, DS‐1205, gefitinib, non‐small cell lung cancer, phase I clinical trial

## Abstract

**Background:**

Tyrosine kinase inhibitors (TKIs) are effective for the treatment of non‐small cell lung cancer (NSCLC) patients with activating mutations of the epidermal growth factor receptor (EGFR), but responses are not durable as tumors develop resistance. DS‐1205c is a novel, specific, orally bioavailable, small‐molecule AXL receptor TKI. In preclinical studies, DS‐1205c restored TKI antitumor activity in a TKI acquired‐resistance *EGFR*‐mutant NSCLC tumor xenograft model.

**Methods:**

This first‐in‐human, multicenter, open‐label Phase 1 study (registered at ClinicalTrials.gov: NCT03599518) primarily evaluated the safety and tolerability of combination therapy with DS‐1205c and gefitinib in Japanese patients with metastatic or unresectable EGFR‐mutant NSCLC and tumor progression during treatment with EGFR‐TKIs. Patients (*n* = 20) received DS‐1205c monotherapy (200–1200 mg twice daily [BID]) in a 7‐day safety monitoring period before combination DS‐1205c/gefitinib (250 mg once daily) in 21‐day cycles.

**Results:**

The observed common treatment‐emergent adverse events (TEAEs) were increased aspartate aminotransferase (35%), increased alanine aminotransferase (30%), rash maculo‐papular (30%), and diarrhea (25%). No serious TEAEs were reported. Plasma concentrations and pharmacokinetic parameters of DS‐1205a (free form of DS‐1205c) were unaffected by concomitant administration of gefitinib. No patient achieved a complete or partial response and 5 patients (25%) had stable disease.

**Conclusion:**

DS‐1205c was generally safe and well tolerated at all dose levels, but the safety profile of ≤800 mg BID was more favorable than 1200 mg BID. The recommended dose for dose‐expansion cohorts of DS‐1205c in combination therapy with gefitinib was 800 mg BID.

## Graphical abstract

Schematic showing the design of this first‐in‐human multicenter, open‐label Phase 1 study of DS‐1205c in combination with gefitinib in subjects with metastatic or unresectable EGFR‐mutant NSCLC and disease progression during treatment with one or more EGFR‐TKIs (gefitinib, erlotinib, afatinib, dacomitinib, or osimertinib).

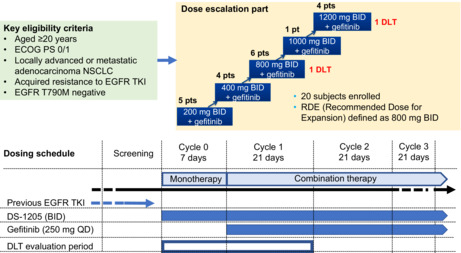

## 1 INTRODUCTION

Established small‐molecule tyrosine kinase inhibitors (TKIs) target epidermal growth factor receptor (EGFR) and are effective in non‐small cell lung cancer (NSCLC) patients with activating and sensitizing EGFR mutations. These mutations are localized in exons 18–21 of the *EGFR* gene and have a prevalence of around 10%–20% in Caucasians with NSCLC adenocarcinomas, and approximately 40%–50% in Asians.^1^, ^2^, ^3^, ^4^ However, responses to TKIs are not durable as tumors develop resistance. The T790M mutation in exon 20 is the most frequent cause (in 50%–60% of cases) of resistance to the first‐ (gefitinib, erlotinib) and second‐generation (afatinib) EGFR TKIs.^1^, ^2^, ^5^ Third‐generation TKIs such as osimertinib are effective against activating EGFR mutations and in NSCLC with the EGFR T790M mutation. However, tumors may acquire resistance to third‐generation TKIs.

AXL is a member of the TAM (TYRO3, AXL, and MERTK) family of receptor tyrosine kinases and, along with its ligand growth arrest‐specific gene 6 (GAS6), plays an important role in promoting growth, survival, and proliferation in both normal and malignant cells.^6^ AXL overexpression in NSCLC and several other solid and hematologic cancers drives a wide range of pathophysiological processes, including epithelial to mesenchymal transition, tumor angiogenesis, decreased antitumor immune response, stem cell maintenance, and resistance to targeted and chemotherapeutic agents.^6^, ^7^, ^8^, ^9^, ^10^ DS‐1205c is a novel, specific, orally bioavailable, small‐molecule inhibitor of AXL which has been developed by Daiichi Sankyo. In pharmacological studies, DS‐1205c was a potent AXL inhibitor with half‐maximal inhibitory concentration (IC_50_) values in the low nanomolar range (1.3–3.7 nM). In in vitro studies, 0.1 μM and 1 μM DS‐1205c in combination with osimertinib (2 μM), slowed the growth of NCI‐H1975 cells, which is a human NSCLC cell line harboring T790M and L858R *EGFR* mutations,^11^ with relative growth rates of 43.1% and 2.2%, respectively. In an *EGFR*‐mutant NSCLC tumor xenograft mouse model, DS‐1205c in combination with gefitinib, erlotinib or osimertinib significantly delayed the onset of tumor resistance to TKIs compared with TKI monotherapy (Daiichi Sankyo, data on file).^12^ Also, DS‐1205c restored antitumor activity of erlotinib in an erlotinib acquired‐resistance *EGFR*‐mutant NSCLC tumor xenograft mouse model.^12^ As the results from these preclinical models demonstrated anti‐tumor activity of DS‐1205c in combination with a TKI in *EGFR*‐mutant NSCLC (T790M‐negative) tumors, we conducted a first‐in‐human Phase 1 study of DS‐1205c in combination with gefitinib in subjects with metastatic or unresectable EGFR‐mutant NSCLC.

## 2 MATERIALS AND METHODS

This was a multicenter, open‐label Phase 1 study of DS‐1205c in combination with gefitinib in subjects with metastatic or unresectable EGFR‐mutant NSCLC and disease progression during treatment with one or more EGFR‐TKIs (gefitinib, erlotinib, afatinib, dacomitinib, or osimertinib). The study was registered at ClinicalTrials.gov (NCT03599518).

### 2.1 Objectives

The primary objective was assessment of the safety and tolerability and determination of the recommended dose of DS‐1205c in combination with gefitinib for the expansion cohort in metastatic or unresectable EGFR‐mutant NSCLC after disease progression during treatment with EGFR‐TKIs.

Secondary objectives were characterization of the pharmacokinetics (PK) of DS‐1205a (free form of DS‐1205c) following DS‐1205c monotherapy, and DS‐1205a plus gefitinib combination treatment; and investigation of the antitumor activity of DS‐1205c in combination with gefitinib.

Exploratory objectives were evaluation of the effect on corrected QT interval (QTc) of DS‐1205c monotherapy and in combination with gefitinib, and identification of biomarkers that correlate with efficacy or toxicity of the combination of DS‐1205c plus gefitinib.

### 2.2 Study participants

All subjects provided written informed consent. Main inclusion criteria were male or female subjects aged ≥20 years with histologically/cytologically confirmed locally advanced or metastatic adenocarcinoma NSCLC which was not amenable to curative surgery or radiation, with Eastern Cooperative Oncology Group (ECOG) performance status (PS) of 0 or 1, and meeting a clinical definition of acquired resistance to EGFR TKI.^13^ Criteria for acquired EGFR TKI resistance included a tumor harboring an EGFR mutation (including G719X, exon 19 deletion, L858R, L861Q) known to be associated with EGFR TKI sensitivity, or experienced clinical benefit from continuous EGFR TKI treatment followed by systemic disease progression defined by Response Evaluation Criteria in Solid Tumors (RECIST) version 1.1,^14^ or World Health Organization (WHO) criteria^15^; and received gefitinib, erlotinib, afatinib, dacomitinib, or osimertinib for at least 6 weeks with well‐controlled related toxicities (less than Grade 3 in severity) at the time of screening. Although the final version of the protocol of the current study (A Multicenter, Open‐Label Phase 1 Study of DS‐1205c in Combination with Gefitinib in Subjects with Metastatic or Unresectable EGFR‐Mutant NSCLC, published on 29 July 2019) required the absence of an EGFR T790M mutation, subjects enrolled in the study before the protocol revision who received osimertinib were enrolled regardless of their EGFR T790M mutation status.

Key exclusion criteria included evidence of small cell histology, or combined small cell and non‐small cell histology in an original or screening tumor biopsy performed since progression; previously documented evidence of ALK fusion, ROS1 fusion, BRAF V600E mutation, rearranged during transfection (RET) fusion, human epidermal growth factor receptor 2 (HER2) mutation, or hepatocyte growth factor receptor (MET) exon 14 skipping mutation; treatment with any cytotoxic chemotherapy, investigational agent or other anticancer drug(s) from a previous cancer treatment regimen or clinical study (other than EGFR TKI) within 14 days of the first dose of the current study treatment, or immune checkpoint inhibitor therapy within 30 days of first dose of the current study treatment; major surgery (excluding a vascular access procedure) within 4 weeks of the first dose of the current study treatment; radiotherapy treatment to more than 30% of the bone marrow or with a wide field of radiation within 4 weeks, or palliative radiation therapy within 2 weeks of the first dose of the current study treatment.

### 2.3 Procedure

Dose escalation involved a run‐in period with DS‐1205c monotherapy for 7 days (Cycle 0), followed by combination treatment with DS‐1205c plus gefitinib (Cycle 1 and beyond). DS‐1205c was administered orally at doses of 200, 400, 800, 1000, and 1200 mg twice daily (BID), and in combination with open‐label oral gefitinib 250 mg once daily in 21‐day cycles.

Safety review meetings (SRMs), consisting of the investigators and the sponsor's medical monitors, reviewed the safety data, including dose‐limiting toxicity (DLT) data, to recommend the dose for the next dose‐escalation cohort and the recommended dose for the dose‐expansion cohort. A Bayesian logistic regression model (BLRM) for inferring the probabilities of DLT at each dose level^16^ was applied to guide the recommended dose for the next dose cohort.^17^ The final decision for the dose of the next cohort was made based on the evaluation of all safety and PK data.

### 2.4 Safety

Safety endpoints included treatment‐emergent adverse events (TEAEs), serious adverse events (SAEs), DLT, standard clinical laboratory parameters, vital sign measurements, electrocardiogram parameters, physical examination findings (including ECOG PS), left ventricular ejection fraction (ECHO/MUGA findings), and ophthalmologic and pulmonary findings. Adverse events (AEs) were coded using Medical Dictionary for Regulatory Activities (MedDRA) and MedDRA/J version 21.1.

### 2.5 Pharmacokinetics

PK parameters were calculated for DS‐1205a (free form of DS‐1205c) and gefitinib in plasma using non‐compartmental analysis. For DS‐1205a, maximum plasma concentration (*C*_max_), time to reach maximum plasma concentration (*T*_max_), and area under the plasma concentration–time curve (AUC) during the first 10 h (AUC_10h_) were measured on Days 1 and 7 during Cycle 0, and Day 1 in Cycles 1 and 2 except that AUC_8h_ was estimated on Day 1 in Cycle 1. In addition, trough plasma concentration (*C*_trough_) was measured on Day 7 in Cycle 0, and Day 1 in Cycles 1 and 2. For gefitinib, *C*_max_, *T*_max_, and AUC_8h_ were measured on Day 1 in Cycles 1 and 2, and additionally, AUC_0–24h_ on Day 1 in Cycle 2.

### 2.6 Efficacy endpoints

The primary efficacy endpoint was the overall response rate (ORR), that is, the number of subjects with best objective response (complete response [CR] or partial response [PR]), divided by the number of subjects who participated in the analysis, which was determined by investigator assessment and based on RECIST version 1.1 criteria. Additional efficacy endpoints included percentage change in size of target lesion(s), duration of response (the time from documented tumor response [CR or PR] to disease progression), disease control rate (DCR: the sum of CR, PR, and stable disease [SD] rates), progression‐free survival (PFS), time to response (TTR), and overall survival (OS).

### 2.7 Biomarkers

Blood samples were collected to measure biomarkers including osteopontin, interleukin‐8 (IL‐8) and soluble AXL. Tumor tissue samples were used to assess the presence of EGFR mutations and to analyze other exploratory biomarkers including AXL expression by immunohistochemistry (IHC). IHC staining was performed using a rabbit monoclonal antibody (C89E7) against AXL purchased from Cell Signaling Technology.

### 2.8 Statistical analysis

TEAEs were tabulated by system organ class (SOC) and preferred term (PT). For the other safety and PK endpoints, continuous data were described by mean and standard deviation (SD), median and range, and categorical data were summarized by frequency. The proportionality of the PK parameters, *C*_max_ and AUC_10h_, was evaluated using a power model (see Table 4). For the efficacy endpoints, best overall response was tabulated as a frequency table, and the time‐to‐event data such as duration of response, PFS, and OS was evaluated using Kaplan–Meier method. All analyses were conducted using SAS® Version 9.4 (SAS Institute).

### 2.9 Ethical approval

This study was conducted in compliance with ethical principles with their origin in the Declaration of Helsinki, the International Council for Harmonization (ICH) consolidated Guideline E6 for Good Clinical Practice (GCP) (CPMP/ICH/135/95), and applicable regulatory requirements including the Japanese Ministry of Health, Labor and Welfare Ordinance No. 28 of 27 March, 1997 and/or The Act on Securing Quality, Efficacy and Safety of Pharmaceuticals, Medical Devices, Regenerative and Cellular Therapy Products, Gene Therapy Products, and Cosmetics No. 1 of 25 November, 2014; and other applicable local regulations.

## 3 RESULTS

A total of 20 Japanese patients were enrolled at 8 sites in Japan. The median (range) age of patients was 68.5 (36–82) years. Patients were mainly female (80%), ECOG PS was 0–1, baseline tumor stage was IIIA (5%) or IVA‐IVB (95%), and 25% of the patients had brain metastases. Baseline EGFR mutations included L858R (60%), T790M (35%), exon 19 deletion (35%), and L861Q (5%). All patients (100%) had previously received one or more TKIs including osimertinib (60%), gefitinib (45%), afatinib (35%), and erlotinib (20%); 40% were previously treated with chemotherapy, and 40% had received radiotherapy. Patients received DS‐1205c 200 mg BID (*n* = 5), 400 mg BID (*n* = 4), 800 mg BID (*n* = 6), 1000 mg BID (*n* = 1) or 1200 mg BID (*n* = 4) in Cohorts 1, 2, 3, 4 and 5, respectively (Table 1). After 1 subject in Cohort 4 (1000 mg BID) completed the DLT evaluation period, the SRM reviewed all the available data regarding safety and PK, and the results from BLRM. The SRM recommended to initiate Cohort 5 (1200 mg BID) and the sponsor decided to proceed to 1200 mg BID accordingly.

**TABLE 1:** Demographics and baseline characteristics by DS‐1205c dose

Variable	Cohort 1	Cohort 2	Cohort 3	Cohort 4	Cohort 5	
	200 mg BID (*n* = 5)	400 mg BID (*n* = 4)	800 mg BID (*n* = 6)	1000 mg BID (*n* = 1)	1200 mg BID (*n* = 4)	Total(*N* = 20)
Age (years), median (range)	67.0 (60–74)	69.5 (67–82)	70.0 (36–75)	41.0 (−)	66.0 (52–81)	68.5 (36–82)
Female, *n* (%)	3 (60)	4 (100)	5 (83)	1 (100)	3 (75)	16 (80)
ECOG performance status, *n* (%)						
0	2 (40)	2 (50)	5 (83)	0 (0)	0 (0)	9 (45)
1	3 (60)	2 (50)	1 (17)	1 (100)	4 (100)	11 (55)
Adenocarcinoma (histology), *n* (%)	3 (60)	4 (100)	6 (100)	1 (100)	4 (100)	18 (90)
Adenocarcinoma (cytology), *n* (%)	5 (100)	3 (75)	6 (100)	1 (100)	3 (75)	18 (90)
Tumor stage at study entry, *n* (%)						
IIIA	1 (20)	0 (0)	0 (0)	0 (0)	0 (0)	1 (5)
IVA	3 (60)	3 (75)	3 (50)	0 (0)	3 (75)	12 (60)
IVB	1 (20)	1 (25)	3 (50)	1 (100)	1 (25)	7 (35)
Brain metastasis, *n* (%)	1 (20)	0 (0)	3 (50)	1 (100)	0 (0)	5 (25)
EGFR mutation, *n* (%)						
T790M	2 (40)	0 (0)	4 (67)	1 (100)	0 (0)	7 (35)
L858R	2 (40)	3 (75)	5 (83)	0 (0)	2 (50)	12 (60)
Exon 19 deletion	3 (60)	1 (25)	1 (17)	1 (100)	1 (25)	7 (35)
L861Q	0 (0)	0 (0)	0 (0)	0 (0)	1 (25)	1 (5)
Prior cancer medical therapy, *n* (%)	5 (100)	4 (100)	6 (100)	1 (100)	4 (100)	20 (100)
≥1 EGFR‐TKI	5 (100)	4 (100)	6 (100)	1 (100)	4 (100)	20 (100)
Osimertinib	2 (40)	1 (25)	6 (100)	1 (100)	2 (50)	12 (60)
Afatinib	1 (20)	2 (50)	1 (17)	0 (0)	3 (75)	7 (35)
Erlotinib	1 (20)	0 (0)	3 (50)	0 (0)	0 (0)	4 (20)
Gefitinib	4 (80)	3 (75)	1 (17)	1 (100)	0 (0)	9 (45)
Chemotherapy	0 (0)	2 (50)	3 (50)	1 (100)	2 (50)	8 (40)
Other	2 (40)	1 (25)	0 (0)	0 (0)	2 (50)	5 (25)
Prior cancer medical regimens, median (range)	2.0 (1–4)	2.5 (1–13)	2.5 (1–6)	7.0 (−)	5.0 (1–6)	3.0 (1–13)
Prior radiotherapy, *n* (%)	2 (40)	0 (0)	3 (50)	1 (100)	2 (50)	8 (40)

Abbreviations: BID, twice daily; ECOG, Eastern Cooperative Oncology Group; EGFR, epidermal growth factor receptor; TKI, tyrosine kinase inhibitor.

### 3.1 Safety

Overall, 19 (95%) patients experienced one or more TEAE. No serious TEAEs nor TEAEs associated with death were reported.

TEAEs with DS‐1205c plus gefitinib combination therapy and DS‐1205c monotherapy are summarized in Table 2 by dose. With combination therapy, the most frequently occurring drug‐related TEAEs were increased aspartate aminotransferase (AST) (*n* = 7; 35%), increased alanine aminotransferase (ALT) (*n* = 6; 30%), rash maculo‐papular (*n* = 6; 30%), diarrhea (*n* = 5; 25%), decreased white blood cell count (*n* = 4; 20%), nausea (*n* = 3; 15%), vomiting (*n* = 3; 15%), and dermatitis acneiform (*n* = 3; 15%). Common TEAEs with DS‐1205c monotherapy were increased AST (*n* = 6, 30%), increased ALT (*n* = 6, 30%), decreased white blood cell count (*n* = 3; 15%) and vomiting (*n* = 3; 15%). No TEAEs of special interest (hepatic event, QTcF prolongation, or interstitial lung disease) were observed.

**TABLE 2:** TEAEs by system organ class and preferred term in DS‐1205c/gefitinib combination therapy and DS‐1205c monotherapy in the safety analysis set (*N* = 20). Data are expressed as *n* (%).

	DS‐1205c/gefitinib combination therapy						DS‐1205c monotherapy					
	200 mg BID (*n* = 5)	400 mg BID (*n* = 4)	800 mg BID (*n* = 6)	1000 mg BID (*n* = 1)	1200 mg BID (*n* = 4)	Total (*N* = 20)	200 mg BID (*n* = 5)	400 mg BID (*n* = 4)	800 mg BID (*n* = 6)	1000 mg BID (*n* = 1)	1200 mg BID (*n* = 4)	Total (*N* = 20)
Subjects with any TEAEs	5 (100)	4 (100)	6 (100)	0 (0)	4 (100)	19 (95)	4 (80)	1 (25)	5 (83)	0 (0)	4 (100)	14 (70)
System organ class and preferred term												
Blood and lymphatic system disorders	0 (0)	0 (0)	1 (17)	0 (0)	1 (25)	2 (10)	0 (0)	0 (0)	1 (17)	0 (0)	1 (25)	2 (10)
Anemia	0 (0)	0 (0)	0 (0)	0 (0)	1 (25)	1 (5)	0 (0)	0 (0)	0 (0)	0 (0)	1 (25)	1 (5)
Neutropenia	0 (0)	0 (0)	1 (17)	0 (0)	0 (0)	1 (5)	0 (0)	0 (0)	1 (17)	0 (0)	0 (0)	1 (5)
Ear and labyrinth disorders	1 (20)	0 (0)	0 (0)	0 (0)	0 (0)	1 (5)	1 (20)	0 (0)	0 (0)	0 (0)	0 (0)	1 (5)
Vertigo	1 (20)	0 (0)	0 (0)	0 (0)	0 (0)	1 (5)	1 (20)	0 (0)	0 (0)	0 (0)	0 (0)	1 (5)
Eye disorders	0 (0)	2 (50)	1 (17)	0 (0)	0 (0)	3 (15)						
Conjunctival hemorrhage	0 (0)	1 (25)	0 (0)	0 (0)	0 (0)	1 (5)						
Dry eye	0 (0)	1 (25)	0 (0)	0 (0)	0 (0)	1 (5)						
Eye disorder	0 (0)	0 (0)	1 (17)	0 (0)	0 (0)	1 (5)						
Gastrointestinal disorders	4 (80)	3 (75)	3 (50)	0 (0)	3 (75)	13 (65)	2 (40)	1 (25)	2 (33)	0 (0)	2 (50)	7 (35)
Diarrhea	0 (0)	3 (75)	1 (17)	0 (0)	1 (25)	5 (25)						
Nausea	1 (20)	0 (0)	0 (0)	0 (0)	2 (50)	3 (15)	1 (20)	0 (0)	0 (0)	0 (0)	1 (25)	2 (10)
Vomiting	2 (40)	0 (0)	1 (17)	0 (0)	0 (0)	3 (15)	2 (40)	0 (0)	1 (17)	0 (0)	0 (0)	3 (15)
Constipation	2 (40)	0 (0)	0 (0)	0 (0)	0 (0)	2 (10)						
Dyspepsia	1 (20)	0 (0)	0 (0)	0 (0)	1 (25)	2 (10)						
Stomatitis	1 (20)	1 (25)	0 (0)	0 (0)	0 (0)	2 (10)	0 (0)	1 (25)	0 (0)	0 (0)	0 (0)	1 (5)
Abdominal pain lower	0 (0)	0 (0)	1 (17)	0 (0)	0 (0)	1 (5)	0 (0)	0 (0)	1 (17)	0 (0)	0 (0)	1 (5)
Abdominal pain upper	0 (0)	0 (0)	0 (0)	0 (0)	1 (25)	1 (5)	0 (0)	0 (0)	0 (0)	0 (0)	1 (25)	1 (5)
General disorders and administration site conditions	1 (20)	0 (0)	2 (33)	0 (0)	1 (25)	4 (20)	1 (20)	0 (0)	1 (17)	0 (0)	0 (0)	2 (10)
Pyrexia	1 (20)	0 (0)	1 (17)	0 (0)	1 (25)	3 (15)	1 (20)	0 (0)	0 (0)	0 (0)	0 (0)	1 (5)
Malaise	0 (0)	0 (0)	1 (17)	0 (0)	0 (0)	1 (5)	0 (0)	0 (0)	1 (17)	0 (0)	0 (0)	1 (5)
Pain	0 (0)	0 (0)	1 (17)	0 (0)	0 (0)	1 (5)						
Hepatobiliary disorders	0 (0)	0 (0)	0 (0)	0 (0)	1 (25)	1 (5)	0 (0)	0 (0)	0 (0)	0 (0)	1 (25)	1 (5)
Hepatic function abnormal	0 (0)	0 (0)	0 (0)	0 (0)	1 (25)	1 (5)	0 (0)	0 (0)	0 (0)	0 (0)	1 (25)	1 (5)
Infections and infestations	2 (40)	1 (25)	0 (0)	0 (0)	1 (25)	4 (20)	2 (40)	0 (0)	0 (0)	0 (0)	0 (0)	2 (10)
Upper respiratory tract infection	1 (20)	0 (0)	0 (0)	0 (0)	1 (25)	2 (10)	1 (20)	0 (0)	0 (0)	0 (0)	0 (0)	1 (5)
Nasopharyngitis	0 (0)	1 (25)	0 (0)	0 (0)	0 (0)	1 (5)						
Paronychia	1 (20)	0 (0)	0 (0)	0 (0)	0 (0)	1 (5)	1 (20)	0 (0)	0 (0)	0 (0)	0 (0)	1 (5)
Injury, poisoning and procedural complications	0 (0)	0 (0)	1 (17)	0 (0)	0 (0)	1 (5)						
Fall	0 (0)	0 (0)	1 (17)	0 (0)	0 (0)	1 (5)						
Investigations	3 (60)	2 (50)	4 (67)	0 (0)	2 (50)	11 (55)	1 (20)	1 (25)	4 (67)	0 (0)	2 (50)	8 (40)
Aspartate aminotransferase increased	2 (40)	1 (25)	2 (33)	0 (0)	2 (50)	7 (35)	1 (20)	1 (25)	2 (33)	0 (0)	2 (50)	6 (30)
Alanine aminotransferase increased	1 (20)	1 (25)	2 (33)	0 (0)	2 (50)	6 (30)	1 (20)	1 (25)	2 (33)	0 (0)	2 (50)	6 (30)
White blood cell count decreased	0 (0)	0 (0)	3 (50)	0 (0)	1 (25)	4 (20)	0 (0)	0 (0)	3 (50)	0 (0)	0 (0)	3 (15)
Electrocardiogram QT prolonged	0 (0)	0 (0)	2 (33)	0 (0)	0 (0)	2 (10)	0 (0)	0 (0)	1 (17)	0 (0)	0 (0)	1 (5)
Neutrophil count decreased	0 (0)	0 (0)	1 (17)	0 (0)	1 (25)	2 (10)	0 (0)	0 (0)	1 (17)	0 (0)	0 (0)	1 (5)
Amylase increased	1 (20)	0 (0)	0 (0)	0 (0)	0 (0)	1 (5)	1 (20)	0 (0)	0 (0)	0 (0)	0 (0)	1 (5)
Blood alkaline phosphatase increased	0 (0)	0 (0)	1 (17)	0 (0)	0 (0)	1 (5)	0 (0)	0 (0)	1 (17)	0 (0)	0 (0)	1 (5)
Blood creatine phosphokinase increased	0 (0)	1 (25)	0 (0)	0 (0)	0 (0)	1 (5)						
Lipase increased	0 (0)	0 (0)	1 (17)	0 (0)	0 (0)	1 (5)	0 (0)	0 (0)	1 (17)	0 (0)	0 (0)	1 (5)
Weight decreased	1 (20)	0 (0)	0 (0)	0 (0)	0 (0)	1 (5)						
Metabolism and nutrition disorders	3 (60)	0 (0)	0 (0)	0 (0)	0 (0)	3 (15)	1 (20)	0 (0)	0 (0)	0 (0)	0 (0)	1 (5)
Decreased appetite	1 (20)	0 (0)	0 (0)	0 (0)	0 (0)	1 (5)						
Hypertriglyceridaemia	1 (20)	0 (0)	0 (0)	0 (0)	0 (0)	1 (5)	1 (20)	0 (0)	0 (0)	0 (0)	0 (0)	1 (5)
Hyperuricaemia	1 (20)	0 (0)	0 (0)	0 (0)	0 (0)	1 (5)						
Musculoskeletal and connective tissue disorders	0 (0)	1 (25)	0 (0)	0 (0)	0 (0)	1 (5)						
Neck pain	0 (0)	1 (25)	0 (0)	0 (0)	0 (0)	1 (5)						
Neoplasms benign, malignant and unspecified (incl cysts and polyps)	1 (20)	1 (25)	1 (17)	0 (0)	0 (0)	3 (15)	1 (20)	0 (0)	0 (0)	0 (0)	0 (0)	1 (5)
Tumor pain	1 (20)	1 (25)	1 (17)	0 (0)	0 (0)	3 (15)	1 (20)	0 (0)	0 (0)	0 (0)	0 (0)	1 (5)
Nervous system disorders	2 (40)	0 (0)	0 (0)	0 (0)	0 (0)	2 (10)						
Hypoaesthesia	1 (20)	0 (0)	0 (0)	0 (0)	0 (0)	1 (5)						
Vagus nerve disorder	1 (20)	0 (0)	0 (0)	0 (0)	0 (0)	1 (5)						
Respiratory, thoracic and mediastinal disorders	0 (0)	1 (25)	0 (0)	0 (0)	0 (0)	1 (5)						
Cough	0 (0)	1 (25)	0 (0)	0 (0)	0 (0)	1 (5)						
Skin and subcutaneous tissue disorders	3 (60)	3 (75)	5 (83)	0 (0)	3 (75)	14 (70)	1 (20)	0 (0)	2 (33)	0 (0)	0 (0)	3 (15)
Rash maculo‐papular	1 (20)	0 (0)	3 (50)	0 (0)	2 (50)	6 (30)	0 (0)	0 (0)	1 (17)	0 (0)	0 (0)	1 (5)
Dermatitis acneiform	0 (0)	3 (75)	0 (0)	0 (0)	0 (0)	3 (15)						
Pruritus	1 (20)	0 (0)	1 (17)	0 (0)	0 (0)	2 (10)	1 (20)	0 (0)	0 (0)	0 (0)	0 (0)	1 (5)
Rash	0 (0)	0 (0)	1 (17)	0 (0)	1 (25)	2 (10)	0 (0)	0 (0)	1 (17)	0 (0)	0 (0)	1 (5)
Dry skin	0 (0)	1 (25)	0 (0)	0 (0)	0 (0)	1 (5)						
Skin hyperpigmentation	1 (20)	0 (0)	0 (0)	0 (0)	0 (0)	1 (5)						

Abbreviations: BID, twice daily; TEAE, treatment‐emergent adverse event.

One subject in Cohort 5 had a TEAE (Grade 2 rash) associated with drug withdrawal. No subjects had TEAEs associated with dose reduction. Seven patients (35%) had TEAEs associated with dose interruption. Of the 19 evaluable patients, DLT was experienced in two patients: one patient in Cohort 3 (800 mg BID) with Grade 3 neutropenia and one in Cohort 5 (1200 mg BID) with Grade 3 nausea, respectively.

TEAEs were Grade 1 (*n* = 6, 30%), Grade 2 (*n* = 7, 35%) or Grade 3 (*n* = 6, 30%). No Grade 4 or 5 TEAEs were observed. Grade 3 TEAEs were observed in 1 patient in Cohort 1 (200 mg BID), which was nausea; in 2 patients in Cohort 3 (800 mg BID), which were increased ALT in 1 patient and decreased white blood cell count and decreased neutrophil count in 1 patient; and in 3 patients in Cohort 5 (1200 mg BID), which were abnormal hepatic function in 1 patient, increased ALT in 1 patient, and nausea in 1 patient. Grade 3 or more TEAEs are summarized in Table 3.

**TABLE 3:** TEAEs (≥Grade 3) by system organ class and preferred term in DS‐1205c/gefitinib combination therapy and DS‐1205c monotherapy in the safety analysis set (*N* = 20). Data are expressed as *n* (%).

	DS‐1205c/gefitinib combination therapy						DS‐1205c monotherapy					
	200 mg BID (*n* = 5)	400 mg BID (*n* = 4)	800 mg BID (*n* = 6)	1000 mg BID (*n* = 1)	1200 mg BID (*n* = 4)	Total (*N* = 20)	200 mg BID (*n* = 5)	400 mg BID (*n* = 4)	800 mg BID (*n* = 6)	1000 mg BID (*n* = 1)	1200 mg BID (*n* = 4)	Total (*N* = 20)
Subjects with any TEAEs (≥Grade 3)	1 (20)	0 (0)	2 (33)	0 (0)	3 (75)	6 (30)	1 (20)	0 (0)	2 (33)	0 (0)	3 (75)	6 (30)
System organ class and preferred term												
Blood and lymphatic system disorders	0 (0)	0 (0)	1 (17)	0 (0)	0 (0)	1 (5)	0 (0)	0 (0)	1 (17)	0 (0)	0 (0)	1 (5)
Neutropenia	0 (0)	0 (0)	1 (17)	0 (0)	0 (0)	1 (5)	0 (0)	0 (0)	1 (17)	0 (0)	0 (0)	1 (5)
Gastrointestinal disorders	1 (20)	0 (0)	0 (0)	0 (0)	2 (50)	3 (15)	1 (20)	0 (0)	0 (0)	0 (0)	1 (25)	2 (10)
Diarrhea	0 (0)	0 (0)	0 (0)	0 (0)	1 (25)	1 (5)						
Nausea	1 (20)	0 (0)	0 (0)	0 (0)	1 (25)	2 (10)	1 (20)	0 (0)	0 (0)	0 (0)	1 (25)	2 (10)
Hepatobiliary disorders	0 (0)	0 (0)	0 (0)	0 (0)	1 (25)	1 (5)	0 (0)	0 (0)	0 (0)	0 (0)	1 (25)	1 (5)
Hepatic function abnormal	0 (0)	0 (0)	0 (0)	0 (0)	1 (25)	1 (5)	0 (0)	0 (0)	0 (0)	0 (0)	1 (25)	1 (5)
Investigations	0 (0)	0 (0)	3 (50)	0 (0)	1 (25)	4 (20)	0 (0)	0 (0)	3 (50)	0 (0)	1 (25)	4 (20)
Alanine aminotransferase increased	0 (0)	0 (0)	1 (17)	0 (0)	1 (25)	2 (10)	0 (0)	0 (0)	1 (17)	0 (0)	1 (25)	2 (10)
White blood cell count decreased	0 (0)	0 (0)	1 (17)	0 (0)	0 (0)	1 (5)	0 (0)	0 (0)	1 (17)	0 (0)	0 (0)	1 (5)
Neutrophil count decreased	0 (0)	0 (0)	1 (17)	0 (0)	0 (0)	1 (5)	0 (0)	0 (0)	1 (17)	0 (0)	0 (0)	1 (5)

Abbreviations: BID, twice daily; TEAE, treatment‐emergent adverse event.

### 3.2 Pharmacokinetics

**FIGURE 1:**
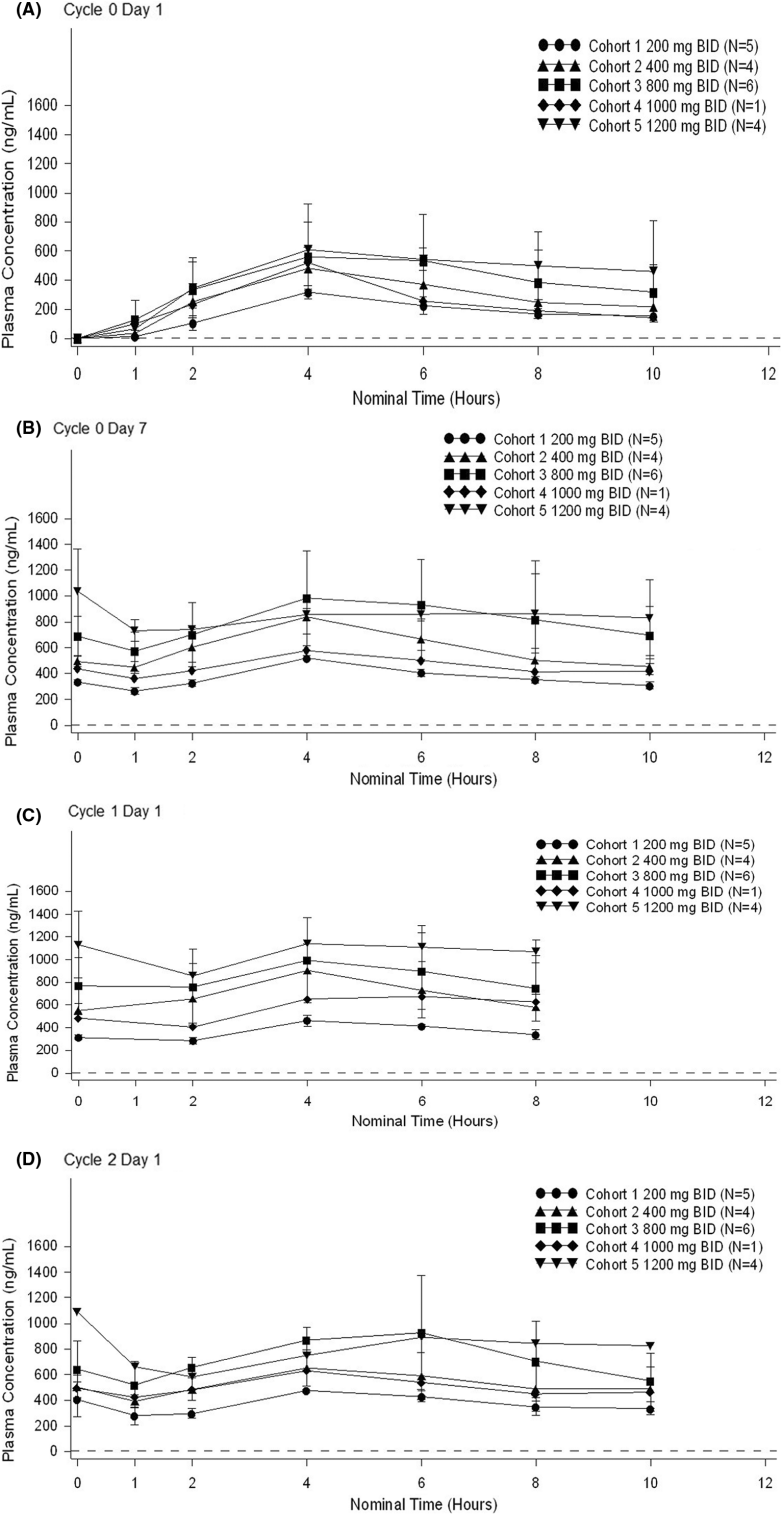
Time course of mean plasma DS‐1205a concentrations for each DS‐1205c dose during DS‐1205c monotherapy (Cycle 0 Day 1 [A] and Cycle 0 Day 7 [B]) and DS‐1205c plus gefitinib combination therapy (Cycle 1 Day 1 [C] and Cycle 2 Day 1 [D]). The lower limit of quantitation (5 ng/ml) is indicated by a dotted line. Error bars show standard deviation.

The lower limit of quantification for DS‐1205a (free form of DS‐1205c) was 5 ng/ml. Time courses of mean plasma DS‐1205a concentrations for each DS‐1205c dose during DS‐1205c monotherapy (Cycle 0, Days 1 and 7) and DS‐1205c plus gefitinib combination therapy (Cycles 1 and 2, Day 1) are shown in Figure 1. For the first DS‐1205c dose (Cycle 0, Day 1), mean DS‐1205a *C*_max_ and AUC_10h_ levels generally increased along with the increasing DS‐1205c dose, reaching a plateau at high dose levels (800–1200 mg BID). By comparing the mean *C*_trough_ values in each cohort on Cycle 0 Day 7, Cycle 1 Day 1, and Cycle 2 Day 1, it was indicated that plasma DS‐1205a concentrations reached steady state within 7 days of twice‐daily administration of DS‐1205c. *C*_trough_ values at the above‐mentioned time points in Cohort 1 were 335, 315, and 408 ng/ml, respectively; 497, 551, and 503 ng/ml, respectively, in Cohort 2; 691, 768, and 643 ng/ml, respectively, in Cohort 3; 437, 486, and 496 ng/ml, respectively, in Cohort 4; and 1040, 1130, and 1090 ng/ml, respectively, in Cohort 5. The PK parameters of DS‐1205a on Day 1 in Cycle 2 were generally similar to those on Day 7 in Cycle 0, suggesting that the PK of DS‐1205a was not affected by gefitinib (Table 4).

**TABLE 4:** Pharmacokinetics of DS‐1205a (free form of DS‐1205c) for DS‐1205c monotherapy (Cycle 0) and DS‐1205c/gefitinib combination therapy (Cycles 1 and 2)

Variable	Cohort 1	Cohort 2	Cohort 3	Cohort 4	Cohort 5
	200 mg BID (*n* = 5)	400 mg BID (*n* = 4)	800 mg BID (*n* = 6)	1000 mg BID (*n* = 1)	1200 mg BID (*n* = 4)
Cycle 0 Day 1, *n*	4	4	6	1	4
*C*_max_ (ng/ml), mean (SD)	316 (46.1)	500 (141)	623 (251)	521 (−)	779 (280)
*T*_max_ (h), median (range)	3.99 (3.92–4.05)	4.99 (3.97–5.93)	3.99 (2.02–6.08)	4.15 (−)	5.08 (4.00–9.80)
AUC_10h_ (ng · h/ml), mean (SD)	1710 (170)	2800 (675)	3860 (1730)	2440 (−)	4280 (893)
Cycle 0 Day 7, *n*	3	4	4	1	2
*C*_max_ (ng/ml), mean (SD)	521 (17.1)	838 (130)	1120 (407)	580 (−)	1080 (268)
*T*_max_ (h), median (range)	3.97 (3.88–4.02)	4.02 (3.98–4.25)	5.08 (4.02–8.00)	4.02 (4.02–4.02)	1.97 (0.00–3.93)
*C*_trough_ (ng/ml), mean (SD)	335 (16.7)	497 (44.2)	691 (155)	437 (−)	1040 (330)
AUC_10h_ (ng · h/ml), mean (SD)	3760 (136)	5990 (945)	8120 (2520)	4560 (−)	8250 (1060)
Cycle 1 Day 1, *n*	3	4	4	1	2
*C*_max_ (ng/ml), mean (SD)	462 (48.1)	907 (266)	995 (375)	677 (−)	1270 (99.0)
*T*_max_ (h), median (range)	3.90 (3.90–4.00)	4.00 (3.88–6.00)	4.01 (3.97–4.05)	6.03 (6.03–6.03)	3.08 (0.00–6.17)
*C*_trough_ (ng/ml), mean (SD)	315 (21.8)	551 (61.6)	768 (249)	486 (−)	1130 (293)
AUC_8h_ (ng · h/ml), mean (SD)	2980 (165)	5670 (1360)	6820 (2590)	4520 (−)	8390 (340)
Cycle 2 Day 1, *n*	2	4	3	1	1
*C*_max_ (ng/ml), mean (SD)	490 (19.8)	688 (130)	1030 (357)	632 (−)	1090 (−)
*T*_max_ (h), median (range)	1.93 (0.00–3.87)	4.99 (0.00–6.10)	4.13 (3.98–6.02)	4.02 (4.02–4.02)	0.00 (0.00–0.00)
*C*_trough_ (ng/ml), mean (SD)	408 (136)	503 (92.7)	643 (217)	496 (−)	1090 (−)
AUC_10h_ (ng · h/ml), mean (SD)	3780 (536)	5290 (1230)	7350 (2160)	5030 (−)	7650 (−)

Abbreviation: BID, twice daily.

The upper limits of 90% CIs for the slope of the power model were less than 1 for all PK parameters of *C*_max_ and AUC_10h_ (Table 5), suggesting that increases in exposure to DS‐1205a were less dose proportional.

**TABLE 5:** Summary of results for pharmacokinetic parameters using a power model

Day of analysis	*C*_max_ (ng/ml)	AUC_10h_ (ng · h/ml)
	β1 (90% CI)	
Cycle 0 Day 1	0.427 (0.215–0.639)	0.455 (0.235–0.674)
Cycle 0 Day 7	0.342 (0.0978–0.585)	0.384 (0.180–0.587)
Cycle 1 Day 1	0.390 (0.152–0.627)	0.355 (0.130–0.580)

*Note*: Power model: *Y* = *β*0 + *β*1 × *X*, where: *Y* = ln(PK parameter); *β*0 = intercept; *β*1 = slope of the power model; and *X* = ln(DS‐1205c dose).

Although the mean *C*_max_ and AUC of gefitinib varied among the cohorts, no specific pattern was observed on Day 1 in Cycle 1 or Day 1 in Cycle 2 (data not shown).

### 3.3 Efficacy

All 20 patients discontinued the treatments, primarily due to the pathologic progression of the disease defined by RECIST criteria (*n* = 17; 85%) or clinical progression (Cohort 5; *n* = 1, 5%). Two patients, one in Cohort 3 and one in Cohort 5, discontinued the treatments due to the withdrawal of consent for the study and AE (see Safety section), respectively.

No patient achieved a CR or PR (ORR = 0%), 25% of patients had stable disease (*n* = 5) and 70% (*n* = 14) experienced disease progression as the best overall response. The disease control rate was 25% (95% CI: 8.7–49.1). Overall, the median number of cycles initiated was 2 and median (range) treatment duration was 7 weeks (0.6–24.9). Median (range) treatment duration in Cohorts 1, 2, 3, 4, and 5 was 6.86 (3.4–12.9), 7.07 (7.0–13.0), 7.00 (1.9–24.9), 7.00 (range not available due to *n* = 1) and 6.71 (0.6–6.9) weeks, respectively. The median PFS was 6.9 (95% CI: 6.1–7.9) weeks. No reduction in tumor size was observed in any subject. There was no obvious DS‐1205c dose‐relationship for efficacy (Table 6).

**TABLE 6:** Efficacy in patients treated with combination DS‐1205c plus gefitinib

Variable	Cohort 1	Cohort 2	Cohort 3	Cohort 4	Cohort 5s	Total (*N* = 20)
	200 mg BID (*n* = 5)	400 mg BID (*n* = 4)	800 mg BID (*n* = 6)	1000 mg BID (*n* = 1)	1200 mg BID (*n* = 4)	
Best overall response, *n* (%)						
Complete response (CR)	0 (0)	0 (0)	0 (0)	0 (0)	0 (0)	0 (0)
Partial response (PR)	0 (0)	0 (0)	0 (0)	0 (0)	0 (0)	0 (0)
Stable disease (SD)	2 (40)	1 (25)	1 (17)	0 (0)	1 (25)	5 (25)
Progressive disease (PD)	3 (60)	3 (75)	5 (83)	1 (100)	2 (50)	14 (70)
Non‐evaluable	0 (0)	0 (0)	0 (0)	0 (0)	1 (25)	1 (5)
Disease control rate (CR + PR + SD)	2 (40)	1 (25)	1 (17)	0 (0)	1 (25)	5 (25)
PFS (weeks), median (95% CI)	6.7 (3.4–12.1)	7.1 (6.1–12.9)	6.9 (2.9–25.0)	6.7 (NR)	6.9 (6.1–6.9)	6.9 (6.1–7.9)

Abbreviations: BID, twice daily; NR, not reached; PFS, progression‐free survival.

### 3.4 Biomarkers and IHC

Following an initial 1 week of DS‐1205c monotherapy, plasma levels of soluble AXL increased from the baseline level by approximately 1.5‐fold. Although most patients showed increases in soluble AXL plasma levels, increases were not dose dependent and no additional increases were observed during combination therapy with gefitinib. Due to small patient numbers, a correlation between an increase in soluble AXL plasma levels and efficacy was not clear. No significant changes of either IL‐8 or osteopontin from baseline levels in plasma were observed in response to DS‐1205c.

At baseline, immunohistochemical analysis showed that 16 of 17 evaluated tumor cell samples were negative for AXL, and 1 showed AXL‐positive staining in around 60% of tumor cells. In contrast, in the portion of stromal nontumor cells in those samples, AXL‐positive staining was observed in fibroblasts (*n* = 10) and immune cells (*n* = 13).

### 3.5 Recommended dose for expansion

Considering all available safety, PK, and exploratory tumor response data, the recommended expansion dose of DS‐1205c in combination with gefitinib was determined to be 800 mg BID.

## 4 DISCUSSION

The primary objective of this first‐in‐human study was to assess the safety and tolerability of DS‐1205c in combination with gefitinib in metastatic or unresectable EGFR‐mutant NSCLC subjects and to determine the recommended dose of DS‐1205c in combination therapy for expansion study. Of the EGFR TKIs, gefitinib was selected because it is known to be generally well tolerated and safety data in Japanese patients have been collected sufficiently.^18^

Of the 20 patients in the Safety Analysis Set, most of them (85%) had at least one TEAE. Increases in transaminases (AST and ALT) were the most commonly reported TEAEs, found in about one‐third of the patients. The frequency of these events tended to increase with increasing doses of DS‐1205c, but no hepatic events (a TEAE of special interest) were observed. No serious TEAEs or TEAEs associated with death were observed in the study.

DS‐1205c in combination with gefitinib was well tolerated, although doses of ≤800 mg BID had a more favorable safety profile compared with the dose of 1200 mg BID. Two patients demonstrated DLT: Grade 3 neutropenia in one patient in Cohort 3 (800 mg BID) and Grade 3 nausea in another in Cohort 5 (1200 mg BID). In addition, one patient in Cohort 5 experienced a TEAE associated with dose interruption, and a second patient in Cohort 5 experienced a TEAE (Grade 2 rash) associated with study discontinuation. Skin reactions, commonly a pustular rash, are very common adverse reactions to gefitinib, but are mainly mild/moderate in severity. Other common adverse reactions include mild/moderate anorexia, diarrhea, vomiting, elevated ALT, nausea, stomatitis and asthenia, which are generally mild in severity. Interstitial lung disease (ILD) is the most serious, relatively common (prevalence of approximately 1%) adverse reaction for gefitinib.^19^, ^20^, ^21^ In this study, no ILD was observed. Similarly, no QTcF prolongation (≥Grade 2), another TEAE of special interest, was observed.

In PK analysis, plasma *C*_max_ and AUC of DS‐1205a (free form of DS‐1205c) generally increased with increasing doses and tended to reach a plateau at high dose levels (800–1200 mg BID). However, a dose‐exposure relationship analysis using power models for *C*_max_ and AUC suggested that increases in exposure to DS‐1205a were not fully dose proportional. Plasma concentrations and PK parameters of DS‐1205a were not affected by the concomitant administration of gefitinib. The overall median treatment duration in the current study was 7 weeks.

No specific pattern was observed in the PK analysis of gefitinib on Day 1 in Cycle 1 or Day 1 in Cycle 2. The PK of gefitinib has been studied in patients with solid tumors^22^, ^23^ and in advanced NSCLC.^24^ In advanced NSCLC, a high day 3/day 8 ratio of plasma trough levels of gefitinib was independently associated with improved PFS.^24^

Five out of 20 patients had stable disease and no patient achieved a complete or partial response. The outcome of stable disease is consistent with preclinical studies showing that DS‐1205c restored the antitumor activity of erlotinib in an erlotinib acquired‐resistance EGFR‐mutant NSCLC tumor xenograft mouse model.^12^ AXL expression level in tumors was also explored. AXL positivity was shown in 1 out of 17 evaluable tumors before DS‐1205c administration. This result was not consistent with previous reports which independently showed about 20% of AXL positivity,^7^, ^25^ probably due to differences in the antibody used for IHC. On the other hand, almost all patients showed an increase of soluble AXL by around 1.5‐fold in plasma during 7 days of DS‐1205c monotherapy. The effect was not dose‐dependent, indicating that soluble AXL achieves a plateau at low DS‐1205c dosages, with soluble AXL possibly being released from normal tissues. Due to insufficient patient numbers, this study was unable to determine a clear correlation between these exploratory outcomes and clinical efficacy. In particular, it would be intriguing to further evaluate the treatment outcome in patients with AXL‐positive tumors as only 1 patient showed tumor AXL positivity in this study. IL‐8 and osteopontin were also evaluated as exploratory pharmacodynamic biomarkers, but neither IL‐8 nor osteopontin showed significant changes from baseline levels following DS‐1205c treatment.

The study has some limitations which reflect the Phase I study design. The sample size was relatively small (20 patients) and, although the study was conducted at 8 sites in Japan, the findings may not be generalizable to the wider population of patients with NSCLC.

In conclusion, DS‐1205c in combination with gefitinib was well tolerated at all dose levels (200–1200 mg BID). However, doses of ≤800 mg BID had a more favorable safety profile compared with 1200 mg BID. Based on the available safety, PK, and exploratory tumor response data, the recommended dose of DS‐1205c in combination with gefitinib was chosen at 800 mg BID. Further studies are needed to confirm the safety profile and evaluate the efficacy of this dose, and potentially to identify predictive biomarkers for responsive patients.

## AUTHOR CONTRIBUTIONS

**Koichi Goto:** Data curation (equal); supervision (equal); writing – original draft (lead); writing – review and editing (lead). **Yoshimasa Shiraishi:** Data curation (supporting); writing – review and editing (equal). **Haruyasu Murakami:** Data curation (equal); supervision (equal); writing – review and editing (equal). **Hidehito Horinouchi:** Data curation (equal); writing – review and editing (equal). **Ryo Toyozawa:** Data curation (equal); writing – review and editing (equal). **Masayuki Takeda:** Data curation (equal); writing – review and editing (equal). **Makiko Uno:** Data curation (equal); writing – review and editing (equal). **Nigel Crawford:** Data curation (equal); writing – review and editing (equal). **Joseph McGill:** Data curation (equal); writing – review and editing (equal). **Takeshi Jimbo:** Data curation (equal); writing – review and editing (equal). **Masato Ishigami:** Data curation (equal); writing – original draft (lead); writing – review and editing (equal). **Gensuke Takayama:** Data curation (equal); writing – review and editing (equal). **Shintaro Nakayama:** Data curation (equal); writing – review and editing (equal). **Shoichi Ohwada:** Data curation (equal); writing – review and editing (equal). **Makoto Nishio:** Data curation (equal); writing – review and editing (equal).

## FUNDING INFORMATION

This study was funded by Daiichi‐Sankyo Co., Ltd.

## CONFLICT OF INTEREST

KG has received lecture fees or honoraria from Eli Lilly Japan K.K., Chugai Pharmaceutical Co., Ltd., Amgen Inc., and Takeda Pharmaceutical Co., Ltd. and research funds from Amgen Inc., AstraZeneca K.K., Boehringer Ingelheim Japan, Inc., Bristol‐Myers Squibb K.K., Chugai Pharmaceutical Co., Ltd., Daiichi Sankyo Co., Ltd., Eisai Co., Ltd., Eli Lilly Japan K.K., Haihe Biopharma Co., Ltd., Ignyta, Inc., Janssen Pharmaceutical K.K., Kissei Pharmaceutica Co., Ltd., Kyowa Kirin Co., Ltd., Life Technologies Japan Ltd., Loxo Oncology, Inc., Medical & Biological Laboratories Co., Ltd., Merck Biopharma Co., Ltd., Merus N.V., MSD K.K., NEC Corporation., Ono Pharmaceutical Co., Ltd., Pfizer Japan Inc., Sumitomo Dainippon Pharma Co., Ltd., Spectrum Pharmaceuticals, Inc., Sysmex Corporation., Taiho Pharmaceutical Co., Ltd., Takeda Pharmaceutical Co., Ltd., and Turning Point Therapeutics, Inc. YS has received research funds from Chugai Pharma. HM has received lecture fees or honoraria from AstraZeneca and Chugai Pharma. HH has received lecture fees or honoraria from AstraZeneca, MSD, Eli Lilly, Ono, BMS, Chugai, Roche, Kyowa‐Kirin, and Novartis; and has participated on Advisory Boards for AstraZeneca, Eli Lilly, Chugai, Roche, Ono, BMS, and MSD. MT has received lecture fees or honoraria from Chugai Pharmaceutical Co. Ltd., AstraZeneca K.K., Bristol‐Myers Squibb, Novartis Pharma K.K., and Ono Pharmaceutical Co. Ltd. MN has received research funds from Ono Pharmaceutical, Bristol‐Myers Squibb, Pfizer, Chugai Pharmaceutical, Eli Lilly. JM has received annual value of remuneration from Daiichi Sankyo Inc.

KG is an Associate Editor of *Cancer Science*.

## ETHICS STATEMENT

All patients provided written informed consent. The protocol, informed consent forms, and any amendments were approved by the institutional review boards of each hospital. The study was registered at ClinicalTrials.gov: NCT03599518.

## Data Availability

The data that support the findings of this study are available from the corresponding author upon reasonable request.
